# Pancreatic head carcinoma derived from the dorsal pancreas is more likely to metastasize early than from the ventral pancreas through microvascular invasion

**DOI:** 10.1097/MD.0000000000039296

**Published:** 2024-08-16

**Authors:** Yuan Gao, Yuhang Shen, Jun Dong, Yang Zhou, Chunfu Zhu, Qiang Yu, Xihu Qin

**Affiliations:** a The Institute of Hepatobiliary and Pancreatic Diseases, The Affiliated Changzhou Second People’s Hospital of Nanjing Medical University, Changzhou Medical Center, Nanjing Medical University, Changzhou, P.R. China; b Department of Hepato-biliary-pancreatic Surgery, The Affiliated Changzhou Second People’s Hospital of Nanjing Medical University, Changzhou Medical Center, Nanjing Medical University, Changzhou, P.R. China; c Department of Pathology, Changzhou Second People’s Hospital, Changzhou Medical Center, Nanjing Medical University, Changzhou, P.R. China.

**Keywords:** overall survival time, pancreatic head cancer, the dorsal pancreas, the ventral pancreas

## Abstract

The development of the pancreatic head originates from the fusion of the ventral and dorsal pancreatic primordia during embryonic development. Theoretically, the origin of pancreatic head cancer also exists from the ventral pancreas and the dorsal pancreas. Among 49 patients with pancreatic head cancer, pancreatic head cancer was divided into pancreatic head cancer originating from the ventral (PHCv) or dorsal pancreas (PHCd) through imaging and pathological classification. The clinical data was collected and compared between the PHCv group and the PHCd group. The results showed that the patients from the PHCd group had worse long-term survival than those from the PHCv group (10 months vs 14.5 months). Similarly, the progression-free survival (PFS) results also indicate that patients from the PHCd group had a shorter time than those from the PHCv group (5 months vs 9.5 months). Further stratified analysis of potentially related factors showed that microvascular invasion is related to poor prognosis, and patients with pancreatic head cancer derived from the dorsal pancreas are more likely to develop microvascular invasion.

## 1. Introduction

Pancreatic ductal adenocarcinoma is an increasing cancer with a less than 6% 5-year survival rate.^[1]^ Among the pancreatic ductal adenocarcinoma, Pancreatic head cancer accounts for almost 75% of pancreatic cancer.^[2]^ A wide *en bloc* pancreaticoduodenal resection has been suggested as the only potential strategy for a potential cure, but the early recurrence is still unavoided. Many clinical studies demonstrated that significant heterogeneity exists in the prognosis of pancreatic head cancer, which underwent the Whipple procedure, with an overall survival range from 6.2 months to 20.9 months.^[3,4]^

In recent years, molecular sequencing has begun to classify pancreatic head cancer subtypes, such as KRAS, TP53, CDKN2A, SMAD4, and ARID1A gene mutation.^[5]^ The development of this molecular biology can better help the research and development of new targeted drugs for pancreatic head cancer and provide a new strategy for exploring the comprehensive treatment of different subtypes of pancreatic head cancer. However, from the perspective of tissue and embryonic development, published literature has reported that the formation of the pancreatic head originates from the fusion of the ventral and the dorsal pancreatic primordium.^[6,7]^ In the pathological anatomy of the adult pancreatic head, the ventral and dorsal pancreas fusion plane can still be found, which can be used to distinguish the ventral and dorsal pancreas of the pancreatic head.^[8]^ From the theoretical analysis of tumorigenesis and development, pancreatic head cancer can also be subtyped into ventral or dorsal-originated pancreatic head cancer. Previous literature and our team’s previous research results advocated that it is feasible to discriminate between pancreatic head cancer originating from the ventral (PHCv) or dorsal pancreas (PHCd).^[9,10]^ However, are there clinical prognostic differences among such subtypes of pancreatic head cancer? So far, there are still no reports.

This study is based on previous reports and discriminates between the PHCv and the PHCd based on the line linking the portal vein (PV) or the superior mesenteric vein (SMV) and the anterior edge of the intrapancreatic bile duct.^[10]^ We reviewed and analyzed the clinical data of these 2 subtypes of pancreatic head cancer and tried to explore the correlation between clinicopathological risk factors and the overall survival time.

## 2. Materials and methods

### 2.1. Patient selection

A total of 49 surgically resected pancreatic head cancer patients were collected between June 2013 and October 2022 at The Affiliated Changzhou Second People’s Hospital of Nanjing Medical University, Changzhou, China. The inclusion criteria: The postoperative pathological diagnosis was pancreatic cancer; The potential line linking the PV/SMV and the anterior edge of the common bile duct could significantly distinguish tumors originating from the ventral pancreas or dorsal pancreas. Complete follow-up data was obtained. On the computerized tomography (CT) scan images, we distinguished the ventral and dorsal pancreas using the main pancreatic duct (the duct of Santorini and the duct of Wirsung), the PV/SMV, and the common bile duct as landmarks. The potential line linking the PV/SMV and the anterior edge of the intrapancreatic bile duct separates the pancreas’s head into the dorsal and ventral pancreas. This study was approved by the ethics committee of The Affiliated Changzhou Second People’s Hospital of Nanjing Medical University, Changzhou Medical Center, Nanjing Medical University, and performed following the statements of the Declaration of Helsinki. All patients provided written informed consent for the use of their pancreatic samples and clinical information. Follow-up time for surviving patients ranged from 1 to 36 months (median, 15.3 months). All 49 patients were followed up for ≥ 1 year after surgery.

### 2.2. Histology and immunohistochemistry (IHC)

All specimens were formalin fixed and paraffin embedded. Immunohistochemical staining with an anti-pancreatic polypeptide (PP) was performed to distinguish the ventral and dorsal pancreas. Histologic sections at 5-μm thickness were stained with hematoxylin-eosin and reviewed by experienced surgical pathologists to determine tumor grade and stage. Tumors were staged according to the eighth edition of the American Joint Committee on Cancer System.^[11]^ IHC was performed using the tissues of patients. The normal pancreatic is extracted from the patients with duodenal malignant tumors who underwent the Whipple procedure. Formalin-fixed and paraffin-embedded tissue slides (5-mm) were deparaffinized and rehydrated. Antigen retrieval was performed by incubating the tissue slides in 0.01 M citric acid buffer at 100°C for 10 minutes. After blocking with 3% H_2_O_2_ and 5% FBS, the slides were incubated with the primary antibodies at 4°C overnight. The next day, The slides were then reacted with polymer HRP reagent. The peroxidase activity was visualized with a diaminobenzidine tetrahydrochloride solution. The sections were counterstained with hematoxylin. Dark brown cytoplasmic staining of ≥ 1% of tumor cells was defined as positive, and no staining or staining of < 1% of cells was defined as negative. The monoclonal antibodies used were as follows: Anti-Pancreatic Polypeptide antibody (1:1000; cat. ab113694; Abcam), Anti-MUC1 antibody (1:1000; cat. ab89492; Abcam), Anti-CD34 (1:1000; cat. ab198395; Abcam), Anti-α-SMA (1:100; cat. ab7817; Abcam).

### 2.3. Statistical analysis

SPSS software (version 23.0; IBM Corp.) was used for statistical analysis. Overall survival was measured from post-operation to death. KM analysis was performed to calculate the overall survival. Start and end points: Follow-up starts after surgery. The endpoint is the date of death from any cause or the date of last follow-up for patients who are still alive at the end of the study. Patient survival curves were drawn using GraphPad Prism 8 software. The difference between survival curves was assessed using the log-rank test. The correlation between the 2 groups and clinicopathological parameters was evaluated by the χ^2^ test and Fisher exact test. Multivariate analysis was used to evaluate the risk factors associated with the 2 groups. Two-tailed *P* values of .05 were considered statistically significant.

## 3. Results

**Figure 1.**
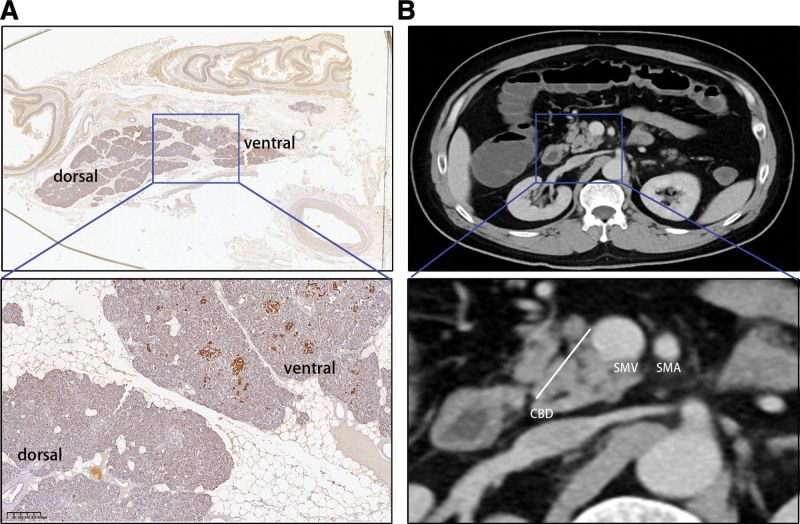
CT scan and IHC distinguishes ventral and dorsal pancreas. (A) Immunohistochemical staining for pancreatic polypeptide in the pancreas head. The ventral pancreas was clearly stained, but the dorsal pancreas was negative. (B) The CT scan image indicates the boundary line between the dorsal and the ventral pancreas. The potential line linking the PV (SMV) and the anterior edge of the intrapancreatic bile duct was verified to divide the subtype groups. CBD = common bile duct, CT = computerized tomography, IHC = Immunohistochemistry, PV = portal vein, SMV = superior mesenteric vein, SMV = superior mesenteric artery.

CT scan and IHC distinguish ventral and dorsal pancreas. According to the theory of differentiation of the ventral and dorsal pancreas, our previous study confirmed that the ventral and dorsal pancreas could be separated by the pancreatic head along the embryological fusion plane through immunohistochemical staining or CT scan.^[9,10]^ To confirm the difference in pathology, we conducted immunohistochemical staining with an anti-pancreatic polypeptide (PP).^[10,12]^ The result demonstrated that PP-positive cells were mainly detected in the ventral pancreas (Fig. 1A). Subsequently, a CT scan was performed to distinguish the ventral and dorsal pancreas by a line linking the PV/SMV and the anterior edge of the common bile duct (Fig. 1B).

**Figure 2.**
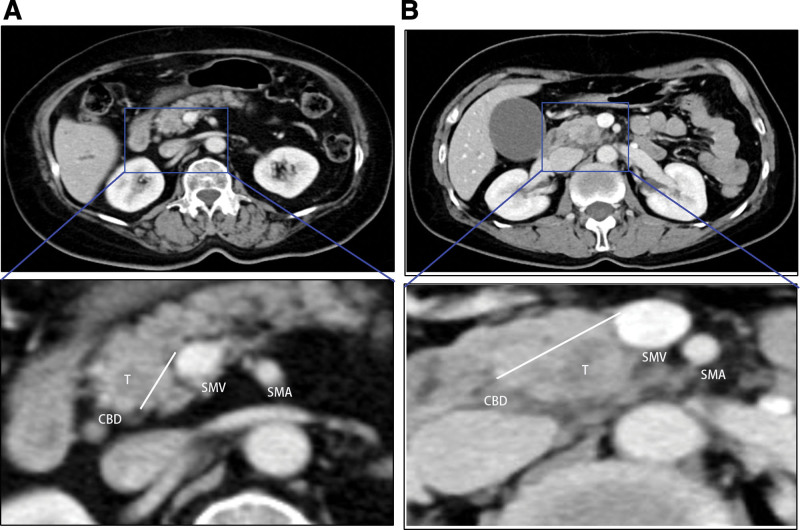
CT scan distinguishes the tumor deriving from dorsal or ventral pancreases. (A) Following the above standard, The CT scan image of pancreatic head cancer arising from dorsal pancreas. (B) The CT scan image of pancreatic head cancer arising from ventral pancreas. CBD = common bile duct, CT = computerized tomography, SMV = superior mesenteric vein, SMV = superior mesenteric artery, T = tumor.

CT scans clarify the pancreatic head adenocarcinoma derived from the dorsal or ventral pancreas. To explore the differences in pancreatic head adenocarcinoma derived from the dorsal or ventral pancreas, a total of 49 patients with pancreatic head adenocarcinoma, including 23 cases in the dorsal pancreas (PHCd group) and 26 cases in the ventral pancreas (PHCv group), were administrated in this retrospective study. According to the above CT standard, we divided that resectable pancreatic head adenocarcinoma into the PHCd group (Fig. 2A) or the PHCv group (Fig. 2B).

**Figure 3.**
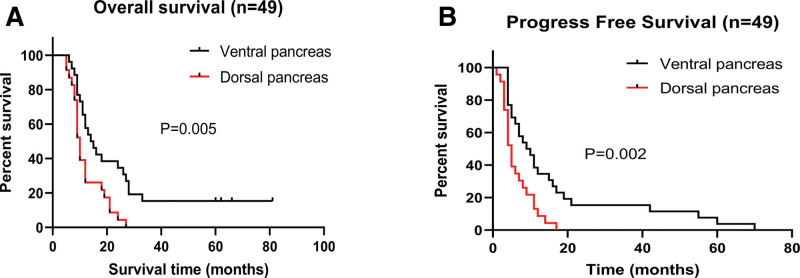
Survival analysis differentiates the prognosis of 2 groups. (A) Kaplan–Meier survival analysis demonstrated the OS of the 49 patients including 23 cases in the dorsal pancreas (PHCd group) and 26 cases in the ventral pancreas (PHCv group). (B) Kaplan–Meier survival analysis demonstrated the PFS of the 49 patients including 23 cases in the dorsal pancreas (PHCd group) and 26 cases in the ventral pancreas (PHCv group). PHCd = pancreatic head cancer originating from the dorsal pancreas, PHCv = pancreatic head cancer originating from the ventral pancreas.

Survival analysis differentiates the prognosis of 2 groups. In the 49 patients who were followed up for at least 13 months, the Kaplan–Meier analysis showed that patients in the PHCv group had a longer overall postoperative survival time with a median OS of 14.5 months than the PHCd group with a median OS of 10.0 months (HR = 0.470, 95% CI: 0.252–0.874) (Fig. 3A; *P* = .005). We also conducted the progression-free survival analysis, and the result demonstrated that patients from the PHCv group had a longer progression-free survival time than those from the PHCd group (Fig. 3B; *P* = .002).

Overall, the results shown in Figures 1 to 3 indicate that patients with pancreatic head adenocarcinoma originating from the dorsal pancreas had a poorer outcome than those from the ventral pancreas.

Clinical characteristics and independent risk factor for poor prognosis between the 2 groups. To explore what kinds of clinical features could correlate with the poorer outcome, we extracted the clinical features from the 2 groups, and univariate analysis was performed to analyze these data. As shown in Table 1, the PHCd group was more frequently associated with diabetes mellitus, TBIL, and microvascular invasion. Subsequently, multivariate analysis was conducted to analyze the above features, and the results show that microvascular invasion was the only independent risk factor associated with survival with a OR value of 4.645 (Table 2; *P* = .026).

**Table 1:** Univariate analysis of clinicopathological differences in patients with pancreatic cancer originating from ventral or dorsal pancreas.

		Ventral pancreas group (n = 26)	Dorsal pancreas group (n = 23)	χ²	*P*
Age (years)		65 ± 11	67 ± 11		.624
Gender	Female	8	11	1.496	.221
	Male	18	12		
BMI (kg/m^2^)		22.79(21.10,25.10)	21.93 ± 3.32		.061
Hypertension	Yes	10	10	0.127	.721
	No	16	13		
Diabetes mellitus	Yes	1	6		.041*
	No	25	17		
TBIL (µmol/L)		124.01 ± 83.03	35.70 (11.70,113.12)		.041*
DBIL (µmol/L)		107.99 ± 76.63	38.10 (4.40,95.36)		.052
ALB (g/L)		39.32 ± 3.76	37.90 ± 3.74		.194
ALT (µ/L)		115.60 (47.45,274.55)	95.10 (38.60,197.10)		.189
AST (µ/L)		83.39 (48.08,168.05)	57.00 (26.60,131.00)		.222
CA-199 (U/mL)		88.77 (43.24,220.63)	188.69 (30.48,403.80)		.400
Pathological grade	G1	0	0	1.793	.181
	G2	14	8		
	G3	12	15		
Tumor stage	I--II	16	12	0.437	.509
	III-IV	10	11		
Microvascular invasion	Yes	5	13	7.302	.007**
	No	21	10		
Nerve invasion	Yes	17	17	0.418	.518
	No	9	6		
Lymph node metastasis	Yes	11	14	1.683	.195
	No	15	9		

* *P* < 0.05, **P < 0.001.

**Table 2:** Multivariate analysis of clinicopathological differences in patients with pancreatic cancer originating from ventral or dorsal pancreas.

	*P*	Odds ratio	95% confidence interval
Diabetes mellitus	.187	4.880	(0.463–51.430)
TBIL	.458	0.998	(0.991–1.004)
Microvascular invasion	.026*	4.645	(1.204–17.925)

* *P* < 0.05, **P < 0.001.

**Figure 4.**
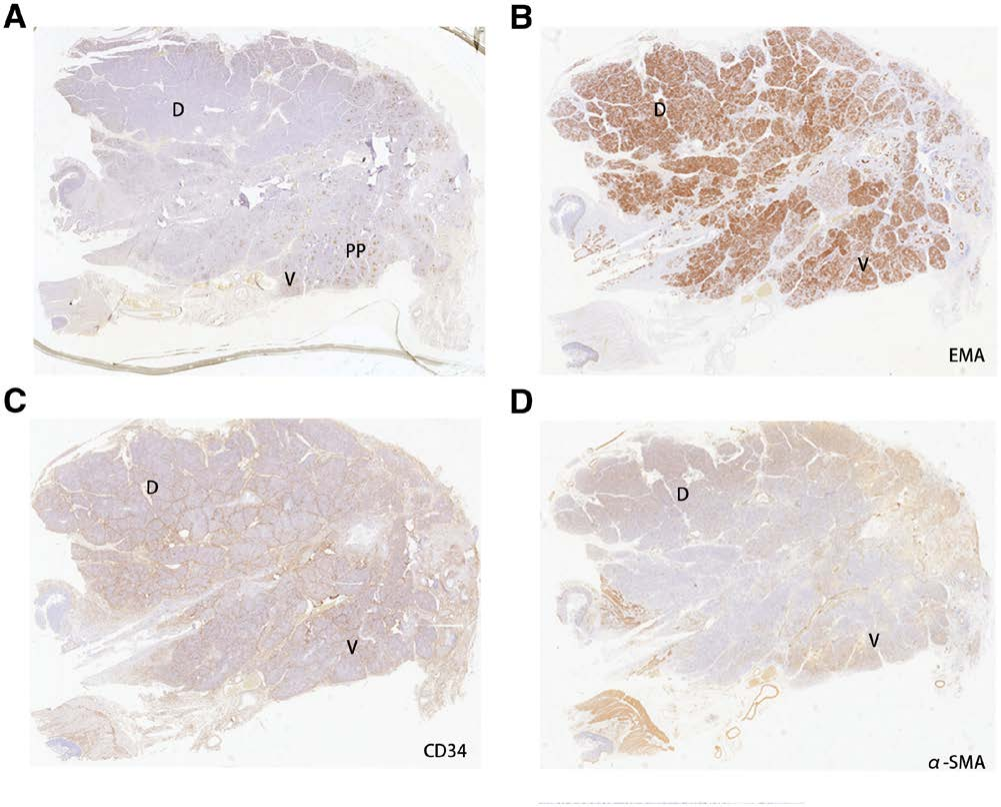
ECM shows no difference between the 2 groups. (A) Pancreatic polypeptide immunohistochemical stain distinguished the ventral pancreas and the dorsal pancreas. (B–D) Immunohistochemical stain of EMA, CD34, and α-SMA in the ventral pancreas and dorsal pancreas. D = the dorsal pancreas, ECM = extracellular matrix, V = the ventral pancreas, PP = pancreatic polypeptide.

**Figure 5.**
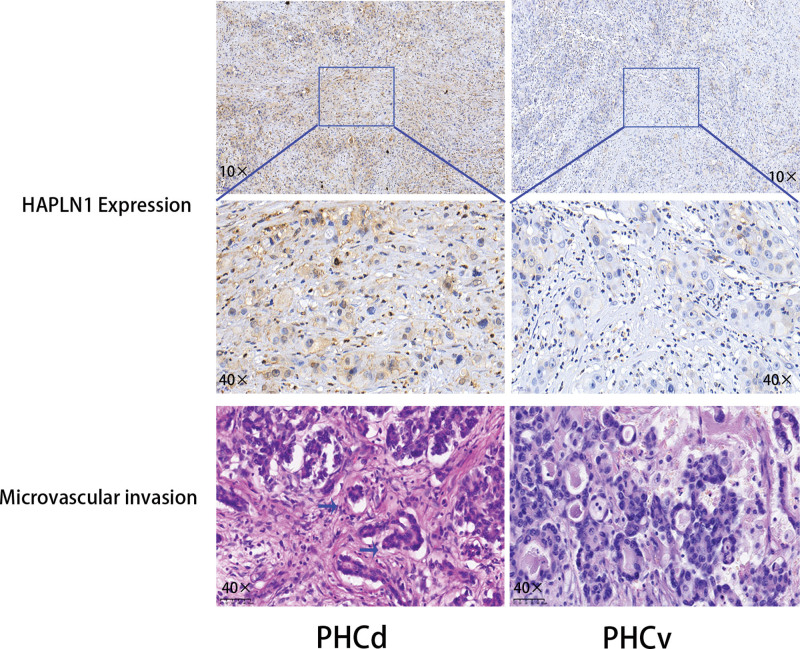
Differential expression of HAPLN1 between the 2 groups. HAPLN1 expression and microvascular invasion in the PHCd group and PHCv group. PHCd = pancreatic head cancer originating from the dorsal pancreas, PHCv = pancreatic head cancer originating from the ventral pancreas. Arrows point to microvascular invasion.

Extracellular matrix shows no difference between the 2 groups. Multiple studies have revealed that the tumor microenvironment contributed to the microvascular invasion and consisted of cancer cells, stromal cells and extracellular components.^[13]^ Among these cells and components, stroma play critical role in connecting tissue cells and remodeling functional tissue. The stroma comprises mesenchymal cells, osteoblasts, the extracellular matrix, etc., and plays an important role in microvascular invasion through physical shield function. Different tissues showed heterogeneity in the stroma; for example, stroma from bone tissue comprises more osteoblasts but less in the pancreas. To further verify if there is a difference between the PHCd group and the PHCv group (Fig. 4A), we conducted IHC to examine the biomarkers of stroma, such as epithelial membrane antigen, CD34, and α-SMA, which are considered as generally accepted biomarkers for examining the difference of stroma in benign or malignant tissues.^[14–16]^ The results demonstrated that there was no difference between the dorsal and ventral pancreas (Fig. 4B–D). Subsequently, we switched to explore the discrepancy between the cancer cells. So, we reviewed multiple published articles and got 6 new oncogenes that are significantly related to the microvascular invasion of pancreatic duct carcinoma, YAP1, ZEB1/2, TWIST1/2, SNAI1, TGF-β, and HAPLN1.^[17–24]^ Following the IHC and HE protocol, we examined the expression of the 6 proteins between the 2 groups. The results showed that only HAPLN1 had a significantly higher expression in the PHCd group than in the PHCv group, microvascular invasion significantly could be seen in the PHCd group (Fig. 5), and the negative data was not shown here.

In sum, the data from Tables 1, 2, and Figures 4–5 revealed that the cancer cell from PHCd group might express more HAPLN1 than PHCv group. More HAPLN1 enhanced cell progression and promoted the capacity of microvascular invasion, and eventually, short the overall survival.

## 4. Discussion

According to the embryonic development of the pancreas, the head of a pancreas consists of the dorsal and ventral pancreas. In recent years, the incidence of pancreatic carcinoma has shown a significant upward trend worldwide; pancreatic head cancer accounts for 70% of pancreatic cancers.^[25,26]^ Although radical pancreaticoduodenectomy combined with extensive lymph node dissection was performed for the patients, the outcome of which was very poor.^[27,28]^ However, further stratified studies showed that there are certain differences in the long-term prognosis between pancreatic head cancer derived from the dorsal pancreas and pancreatic head cancer derived from the ventral pancreas.^[29]^ Our study revealed that microvascular invasion is the major factor contributing to the short overall survival. Microvascular invasion may be affected by pathologic factors: The structural differences of the tissues around the tumor, with irregular loose connective tissue containing spare blood vessels^[30–32]^; The composition of the stroma around the tumor promotes tumor cells to infiltrate into the stroma and then infiltrate microvascular^[33]^; The tumor cells’ biological characteristics and abnormally increased expression of oncogenes that promote tumor cell activity may cause tumor cells to invade microvascular.^[34,35]^ The results of the literature search revealed that HAPLN1 is a tumor protein that has been discovered and confirmed to affect the invasiveness of pancreatic cancer cells.^[17]^ However, this study cannot extract pancreatic head cancer cells derived from the ventral pancreas and those derived from the dorsal pancreas. However, the differential expression of HAPLN1 protein in tissues from different sources of pancreatic head cancer was examined by immunohistochemistry, and the results showed statistical differences. This verifies our above conjecture. Although the above results partially explain the differences in prognosis of pancreatic head cancer from different sources, other possible causes cannot be ruled out, such as congenital differences between the ventral and dorsal pancreatic primordia during pancreatic development.

So far, our study first explored the difference between dorsal pancreas cancer and ventral pancreas cancer. The clinical study revealed microvascular invasion as the core factor that promoted dorsal pancreatic cancer.

## Author contributions

**Conceptualization:** Yuan Gao, Yuhang Shen, Jun Dong, Yang Zhou, Chunfu Zhu, Qiang Yu, Xihu Qin.

**Data curation:** Yuan Gao, Yuhang Shen, Jun Dong, Yang Zhou, Chunfu Zhu, Qiang Yu, Xihu Qin.

**Formal analysis:** Yuan Gao, Yuhang Shen, Jun Dong, Yang Zhou, Chunfu Zhu, Qiang Yu, Xihu Qin.

**Funding acquisition:** Yuan Gao, Yuhang Shen, Jun Dong, Yang Zhou, Qiang Yu, Xihu Qin.

**Investigation:** Yuan Gao, Yuhang Shen, Jun Dong, Yang Zhou, Xihu Qin.

**Methodology:** Yuan Gao, Yuhang Shen, Jun Dong, Yang Zhou, Xihu Qin.

**Project administration:** Yuan Gao, Yuhang Shen, Jun Dong, Yang Zhou, Xihu Qin.

**Resources:** Yuan Gao, Yuhang Shen, Jun Dong, Yang Zhou, Xihu Qin.

**Software:** Yuan Gao, Yuhang Shen, Jun Dong, Yang Zhou, Xihu Qin.

**Supervision:** Yuan Gao, Yuhang Shen, Jun Dong, Yang Zhou, Chunfu Zhu, Xihu Qin.

**Validation:** Yuan Gao, Yuhang Shen, Jun Dong, Xihu Qin.

**Visualization:** Yuan Gao.

**Writing – original draft:** Yuan Gao.

**Writing – review & editing:** Yuan Gao, Chunfu Zhu, Qiang Yu, Xihu Qin.
